# The *Kengyiliahirsuta* karyotype polymorphisms as revealed by FISH with tandem repeats and single-gene probes

**DOI:** 10.3897/compcytogen.v15.i4.71525

**Published:** 2021-11-03

**Authors:** Xiaoyan Tao, Bo Liu, Quanwen Dou

**Affiliations:** 1 Key Laboratory of Adaptation and Evolution of Plateau Biota, Northwest Institute of Plateau Biology, Chinese Academy of Sciences, Xining, China; 2 Key Laboratory of Crop Molecular Breeding, Qinghai Province, Northwest Institute of Plateau Biology, Chinese Academy of Sciences, Xining, China; 3 University of Chinese Academy of Sciences, Beijing, China

**Keywords:** Homoeology, karyotype, *Kengyiliahirsuta*, single-gene FISH

## Abstract

*Kengyiliahirsuta* (Keng, 1959) J. L. Yang, C. Yen et B. R. Baum, 1992, a perennial hexaploidy species, is a wild relative species to wheat with great potential for wheat improvement and domestication. The genome structure and cross-species homoeology of *K.hirsuta* chromosomes with wheat were assayed using 14 single-gene probes covering all seven homoeologous groups, and four repetitive sequence probes 45S rDNA, 5S rDNA, pAs1, and (AAG)_10_ by FISH. Each chromosome of *K.hirsuta* was well characterized by homoeological determination and repeats distribution patterns. The synteny of chromosomes was strongly conserved in the St genome, whereas synteny of the Y and P genomes was more distorted. The collinearity of 1Y, 2Y, 3Y and 7Y might be interrupted in the Y genome. A new 5S rDNA site on 2Y might be translocated from 1Y. The short arm of 3Y might involve translocated segments from 7Y. The 7 Y was identified as involving a pericentric inversion. A reciprocal translocation between 2P and 4P, and tentative structural aberrations in the subtelomeric region of 1PL and 4PL, were observed in the P genome. Chromosome polymorphisms, which were mostly characterized by repeats amplification and deletion, varied between chromosomes, genomes, and different populations. However, two translocations involving a P genome segmental in 3YL and a non-Robertsonial reciprocal translocation between 4Y and 3P were identified in two independent populations. Moreover, the proportion of heterozygous karyotypes reached almost 35% in all materials, and almost 80% in the specific population. These results provide new insights into the genome organization of *K.hirsuta* and will facilitate genome dissection and germplasm utilization of this species.

## Introduction

*Kengyilia* Yen, Yang, C. Yen et J. L. Yang, 1990, is a perennial genus belonging to the tribe Triticeae (family Poaceae), species of which are commonly distributed in central Asia and the Qinghai–Tibetan plateau (Yen and Yang 1990). *Kengyilia* grows in meadows, steppes, the fringes of forests, and also semi deserts or extremely dry deserts at altitudes from 1100 to 4750 m (Yang et al. 1992). Approximately 32 species and subspecies have been documented in this genus (Cai 1999). *Kengyiliahirsuta* (Keng, 1959) J. L. Yang, C. Yen et B. R. Baum, 1992, is a species of *Kengyilia*, which is distributed in Qinghai and Gansu in China. *K.hirsuta* can be found around sandy areas in high altitude regions, owing to its high tolerance to cold hardness and drought, as well as its distinct perennial characteristics. As a grain grass, *K.hirsuta* has potential genetic resources for utilization in cereal crop development.

Many desired genes, such as disease resistance and stress tolerant genes, were introduced to the wheat background using specific cytology techniques involving hybridization with the tertiary gene pool species (Jiang et al. 1994). The transfer of apomixis into wheat was attempted by crossing wheat and *Elymusrectisetus* (Nees, 1846) Á. Löve et Connor, 1982, which is apomictic (Wang et al. 1993; Liu et al. 1994). Perennial crops are thought to have great potential for truly sustainable production systems (Glover et al. 2010). The perennial habit has been transferred into annual wheat by introducing alien chromosomes from related perennial species (Lammer 2004; Abbasi et al. 2020). However, no widely used perennial cultivars were produced following these attempts (Dehaan et al. 2020). Direct domestication of wild perennial grass relatives of wheat, such as *Thinopyrumintermedium* (Host, 1805) Barkworth et Dewey, 1985, is an alternative approach (Dehaan et al. 2020). *K.hirsuta*, which is a close perennial relative of wheat, is charaterized by tall stalks, high seed setting rates, and mild seed shattering in the growing regions, and is a promising perennial grain candidate for domestication in the alpine or wider regions.

*Kengyilia* species were identified by cytological methods as allopolyploids with the genome constitution StPY (Jensen 1990, 1996). The St and P genomes are derived from *Pseudoroegneria* (Nevski, 1934) Á. Löve, 1980, and *Agropyron* Gaertner, 1770, respectively, although the origin of the Y genome is still unknown (Wang et al. 1994). The molecular karyotype of *K.hirsuta* was first characterized by using chromosome identification under florescence *in situ* hybridization (FISH), with probes of 5S rDNA, 45S rDNA, pAs1, and (AAG)_10_ (Dou et al. 2013). However, the cross-species homoeology of the identified chromosomes have not yet been ascertained. Moreover, inter-genomic arrangements significantly affected by the environmental factors were reported in *K.thoroldiana* (Oliver, 1893) J. L. Yang, C. Yen et B. R. Baum, 1992 (Wang et al. 2012) and *Elymusdahuricus* Turcz. ex Griseb. complex (Yang et al. 2017), whereas chromosomal variations between different populations in *K.hirsuta* remain unknown. Determination of chromosomal homoeology is very useful for the discovery and utilization of important genes and alleles in wild relatives. Alien gene transfer by interspecific hybridization is strongly affected by chromosome collinearity (Friebe et al. 1996; Qi et al. 2007). Targeted gene cloning may be facilitated by capturing the syntenic chromosomes of the wild relatives, which include complex genomes, by flow sorting (Tiwari et al. 2015; Said et al. 2018) or even by micro dissection (Sheng et al. 2020; Soares et al. 2020).

In the present study, the homoeology of the *K.hirsuta* chromosomes with those of wheat were determined using single-genes FISH. Further, the polymorphisms of the chromosomes between different populations were investigated. The results will be helpful for exploring genes from *K.hirsuta* by comparison of genomics in the chromosomal levels, and could accelerate the domestication of *K.hirsuta* as a new perennial crop.

## Material and methods

### Plant material

**Figure 1.**
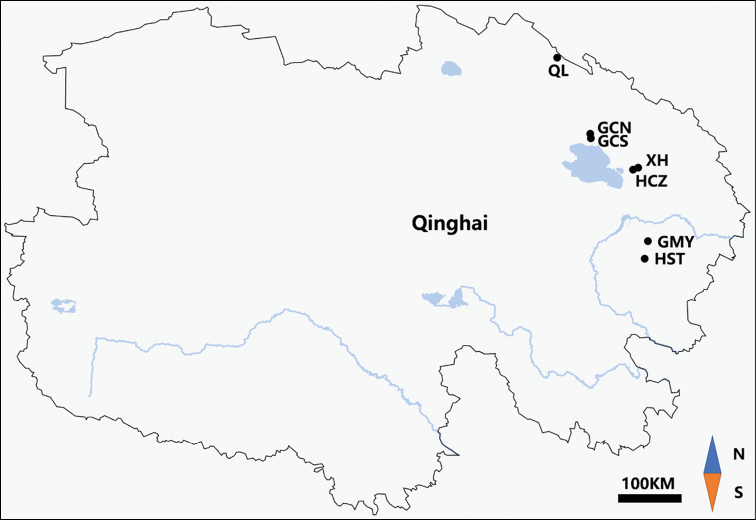
Map of sample collection sites.

The seeds of *K.hirsuta* were collected individually from 7 different locations in Qinghai, China. Three to five individuals were randomly selected for cytological investigation in each population. Detailed information on collection sites is listed in Table 1 and Fig. 1.

**Table 1.** Plant materials used in this study.

Identification ID	Location	Latitude (N), Longitude (E)	Altitude (m)
HST	Guinan, Qinghai	35°31'21"N, 101°6'9.8"E	3370
GMY	Guinan, Qinghai	35°47'28"N, 101°8'51"E	3200
HCZ	Haiyan, Qinghai	36°50'44"N, 100°56'0.9"E	3500
XH	Haiyan, Qinghai	36°58'43"N, 100°54'24"E	3130
GCN	Gangcha, Qinghai	37°21'33"N, 100°8'0.4"E	3350
GCS	Gangcha, Qinghai	37°19'19"N, 100°10'7.6"E	3350
QL	Qilian, Qinghai	38°29'18"N, 99°34'22"E	3450

### Preparation of cDNA sequences

Fourteen cDNA clones were selected for single-gene probes, which were previously mapped to the short and long arms of the seven homoeologous chromosomes in common wheat, respectively, by Danilova et al. (2014) (Table 2). The cDNA clones were developed and kindly supplied by the National BioResource Project-Wheat, Japan. cDNA sequences were amplified with PrimeSTAR Max DNA Polymerase (Takara Bio Inc., Kusatsu, Shiga, Japan Cat. # R001B) and standard primers T3/T7 by polymerase amplification reactions (PCR). The amplification products were purified with an Omega MicroElute Cycle-Pure Kit (Omega Bio-tek, Inc., Norcross, Georgia, USA Cat. # D6293-02).

### Probes labeling

All cDNA probes were labeled by nick translation as described previously, with minor modifications (Kato et al. 2004, 2006). Briefly, all cDNA amplifications were labeled with tetramethyl-rhodamine-5-dUTP (Roche Diagnostics GmbH, Mannheim, Germany Cat. # 11534378910). After the addition of EDTA (500 mM, pH 8.0) to terminate the nick translation reaction, the probes were purified using the Omega DNA Probe Purification Kit (Cat. # D6538-02) following the manufacturer’s recommendations.

The oligonucleotide probes were used for 5S rDNA, 45S rDNA, pAs1, and (AAG)_10._ The designated oligonucleotides pAs1-1 plus pAs1-2, 5Sg, Oligo-pTa71-2 representing pAs1, 5S and 45S rDNA respectively (Danilova et al. 2012; Tang et al. 2014). All oligonucleotides were end-labeled using either fluorescein amidite (FAM; green) or carboxy tetramethyl rhodamine (TAMRA; red) (Sangon Biotech Co., Ltd., Shanghai, China).

Genomic DNAs of *Pseudoroegneriastipifolia* (Nevski, 1934) Á. Löve, 1984 (2*n* = 2*x* = 14; St genome) and *Aropyroncristatum* Gaertner, 1770, (2*n* = 4*x* = 28; P genome) were fragmented by autoclaving following the procedures of Dou et al. (2009). The treated genomic DNAs were labeled with tetramethyl-rhodamine-5-dUTP (red) or fluorescein-12-dUTP (green) (Roche Diagnostics, Germany) by a random primer labeling method.

### Chromosome preparation

The seeds were germinated on moist filter paper in Petri dishes at room temperature. Root tips with a length of 1–2 cm were collected and pretreated with nitrous oxide at a pressure of 7–8 atm for 2 h at room temperature following the method of Kato (1999). The pretreated root tips were fixed in ethanol-glacial acetic acid (3:1, v/v) for at least 30 min at room temperature, subsequently; each root tip was squashed in a drop of 45% acetic acid and observed with a phase contrast microscope (Olympus BX43). The slides with good chromosome spread were selected for further FISH.

### FISH and microphotometry

The chromosome preparations were denatured in 0.2M NaOH and 70% ethanol for 10 minutes at room temperature; subsequently, they were rinsed in cold 70% ethanol for 1 hour and quickly air dried. The hybridization mixture per slide (total volume = 10 μl) contained 100 ng labeled probe DNA, 50% v/v formamide, 2 × SSC, 10% w/v dextran sulfate, and 0.1 μg salmon sperm DNA. The hybridization mixture with single-gene probes or labeled genomic DNAs was denatured in the boiled water for 5 minutes, and immersed in ice-water for at least 10 minutes. The hybridization with oligo-based probes was conducted directly without denaturation. The hybridization was carried out overnight at 37 °C. A sequential FISH technique with multiple rounds of hybridization on the same chromosome preparation was adopted in this study. The first hybridization was conducted with single-gene probes. The slide was washed in 2 × SSC at 42 °C three times in this round, and at least 10 cells with distinct signals were captured. Subsequently, the slide was washed by tap water. The second and third round hybridizations were carried out using the probe combination 45S rDNA and 5S rDNA, and the combination of pAs1 and (AAG)_10_, respectively. After each of the second and third rounds of hybridization, the hybridization signals were removed by heating at 55 °C for 10 minutes on hot plate, followed by washing in tap water. The last round was genomic hybridization *in situ* hybridization (GISH) with genomic DNA probes of *P.stipifolia* and *A.cristatum*. Images were captured with a cooled CCD camera (DP80) under a fluorescence microscope (Olympus BX63). Finally, images were adjusted with Adobe Photoshop 6.0 for contrast and background optimization.

## Results

### Karyotype with homoeology determination

**Figure 2.**
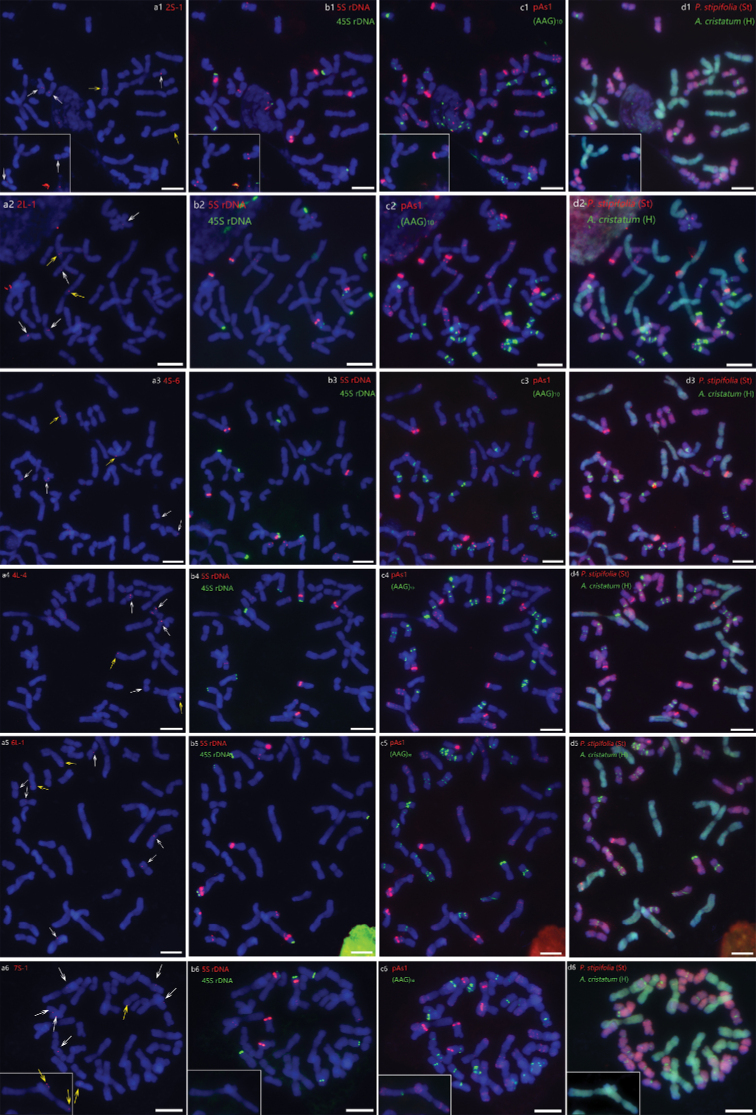
Sequential FISH-GISH on mitotic chromosomes of *K.hirsuta* with single-gene and repetitive sequence probes **a** single-gene probes (arrowed) **b** 45S rDNA (green) and 5S rDNA (red) **c** pAs1 (red) and (AAG)_10_ (green) **d** genomic DNA probes of *P.stipifolia* (red) and *A.cristatum* (green). Scale bars: 10 μm.

Fourteen selected single-gene probes are evenly distributed on the chromosomes of the seven homoeologous groups in wheat, two of which were mapped on the short arm and the long arm on each chromosome, respectively. Thus, two single-gene probes on each chromosome were used in the present study to identify the corresponding species-crossed homoeologous chromosomes in *K.hirsuta*. The individuals of the population HCZ (Table 1) were randomly selected for single-gene mapping. The first round of hybridization using single-gene probes showed that most of the single-gene probes produced the expected six hybridization signals in *K.hirsuta*, each genome of which had two hybridization signals on the pair of the homologous chromosomes (Fig. 2a). Exceptionally, the single-gene probe 6L-1 produced eight signals (Fig. 2 a5), whereas the single-gene probe 7S-1 yielded 10 signals in the chromosomes (Fig. 2 a6). Further, the single-gene mapped chromosomes were clearly identified by hybridizations with 5S rDNA, 45S rDNA, pAs1, and (AAG)_10_ after the second and third rounds of hybridization (Fig. 2b and 2c). Lastly, the characterized target chromosomes were allocated to the different genomes as St, Y and P by GISH (Fig. 2d).

**Figure 3.**
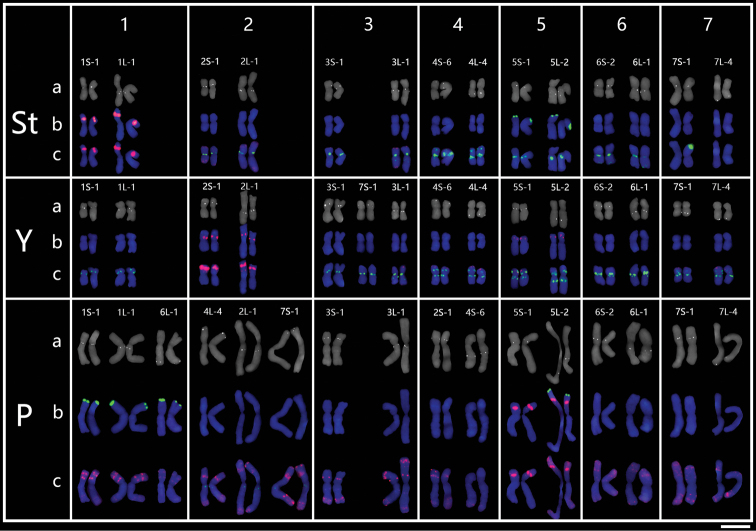
Molecular karyotype of *K.hirsuta* with 14 single-gene probes and repetitive sequences probes **a** single-gene probe **b** 45S rDNA (green) and 5S rDNA (red) **c** pAs1 (red) and (AAG)_10_ (green). Scale bar: 10 μm.

**Figure 4.**
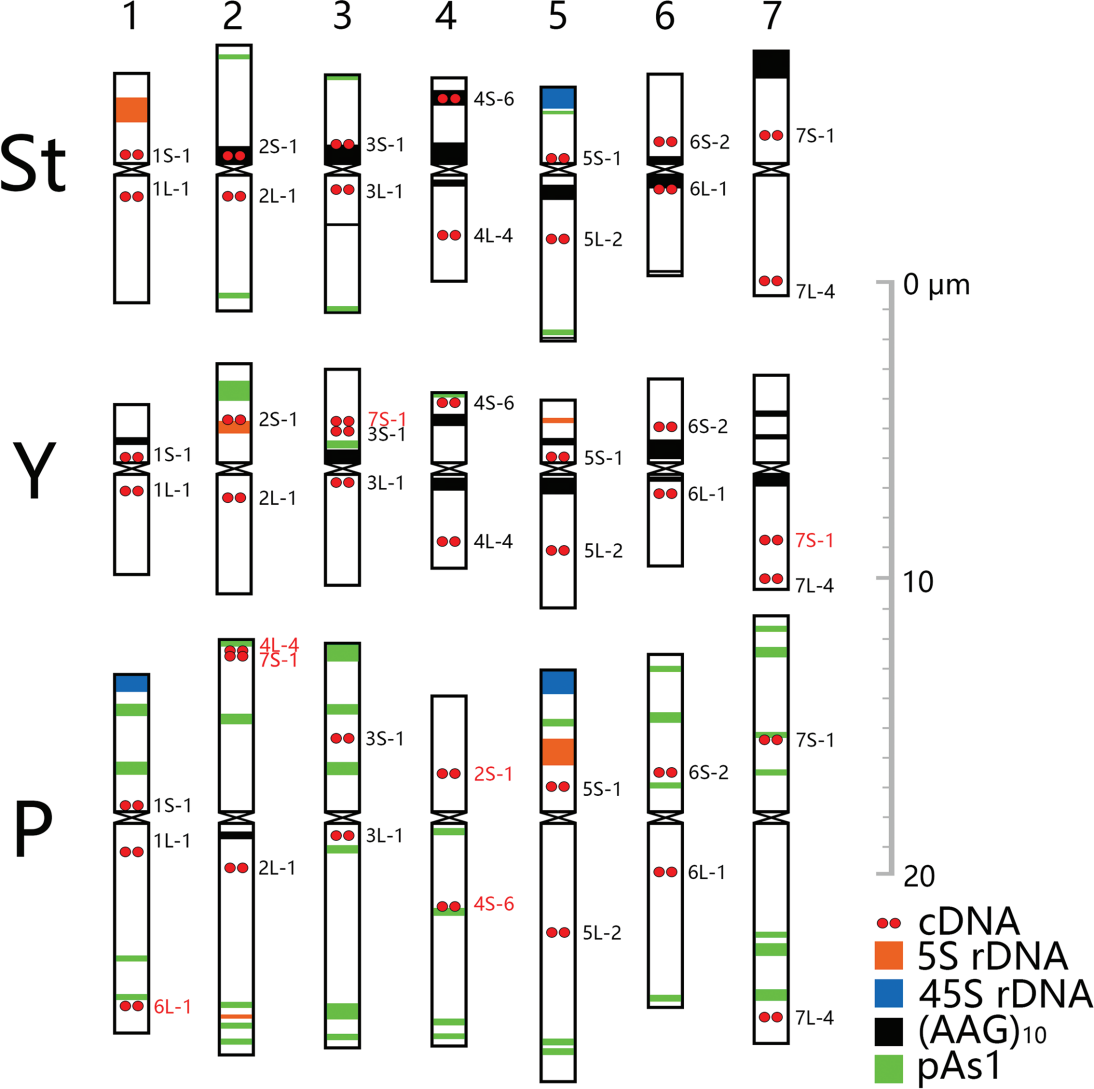
Idiogram for chromosomes of *K.hirsuta* showing the distribution of 45S rDNA, 5S rDNA, pAs1, (AAG)_10_ and single-genes. The names of single-gene probes that hybridized to more than one chromosome, and to non-homoeologous chromosome are highlighted in red. The color scheme (bottom right) shows the color of each probe as represented in this idiogram.

After multiple hybridizations with 14 single-gene probes, each of the *K.hirsuta* chromosomes was not only characterized with distinct chromosomal markers, but also its homoeology was determined (Fig. 3; Table 3). The St genome chromosome sizes ranged from 6.60 to 8.76 μm with an average of 7.68 μm; the Y genome chromosome sizes ranged from 5.54 to 7.56 μm with an average of 6.55 μm; and the P genome chromosome sizes ranged from 11.70 to 14.21 μm with an average of 12.96 μm. The relative chromosome arm ratio (long arm to short arm) ranged from 2.13 for the largest chromosome, 5St, to 1.07 for the smallest, 7St, in the St genome; the corresponding ratios ranged from the 2.08 for the largest chromosome, 5Y, to 1.09 for the smallest, 6Y in the Y genome; and from 1.90 for the largest chromosome, 4P, to 1.12 for the smallest, 7P in the P genome (Table 3). A referenced karyotype idiogram of *K.hirsuta* was suggested with the designated chromosome number from 1–7 corresponding to those in common wheat (Fig. 4). The features of chromosomes in *K.hirsuta* are as follows.

Chromosomes 1St and 5St were distinguished by 5S rDNA and 45S rDNA sites on the short arm, respectively. Both 2St and 6St have one major (AAG)_10_ hybridization site, differing from 3St and 4St which both included two discrete (AAG)_10_ hybridization sites. However, 2St was associated with (AAG)_10_ on the short arm near the centromere, distinct from 6St with (AAG)_10_ around the centromere, while 3St showed an additional (AAG)_10_ minor signals in the long arm rather than 4St showed an additional major signal in the short arm. 7St showed none or one major (AAG)_10_ hybridization site in the telomeric region of the short arm, and thus differed from the others (Fig. 4).

Chromosome 1Y was the smallest, with one major or minor (AAG)_10_ site on the short arm. Chromosomes 2Y and 5Y harbored a 5S rDNA site in the intercalary and subtelomeric regions on the short arm, respectively. Both 3Y and 6Y had one distinct major (AAG)_10_ site around the centromere, but 6Y showed stronger hybridization intensity than 3Y. Chromosome 4Y showed the two (AAG)_10_ sites similar to 5Y, but 5Y was with the 5S sites. Chromosome 7Y exhibited one major (AAG)_10_ site near the centromere on the long arm and one or two minor (AAG)_10_ sites in the intercalary region on the short arm (Fig. 4).

Chromosome 1P was distinct with one major 45S rDNA in the telomeric region of the short arm. Chromosome 2P was identified as a metacentric chromosome with a distinct pAs1 hybridization on the intercalary part of the short arm, and a weak (AAG)_10_ around the centromere. The single gene probe 4L-4 and 2L-1 produced hybridizations on the short and the long arms, respectively, in this chromosome. Chromosome 4P was characterized as a sub-metacentric chromosome with more pAs1 hybridizations on the long arm than on the short arm. The single gene probes 2S-1 and 4S-6 produced hybridizations on the respective short and long arms in this chromosome. Therefore, 2P and 4P could be reciprocal translocation chromosomes. Tentatively, 2P and 4P were respectively designated as 4PL/2PL and 2PS/4PS. Chromosome 3P was identified as a metacentric chromosome with opulent pAs1 hybridizations on the short arm. Chromosome 5P was characterized by the segregated 5S rDNA and 45S rDNA sites on the short arm. Chromosome 6P was characterized by weakly dispersed pAs1 hybridizations mostly distributed in the half of the short arm near the telomere, whereas 7P was identified as including dispersed pAs1 hybridizations on both arms (Fig. 4).

### Synteny between chromosomes of *K.hirsuta* and those of common wheat and *Agropyroncristatum*

Comparison between the single-gene probes on *K.hirsuta* and the homoeologous groups of common wheat showed that most of the probes hybridized to the corresponding homoeologous chromosome arms of *K.hirsuta*, generally in their corresponding positions (Danilova et al. 2014). This indicates the chromosomal synteny across species. However, the degree of synteny varied between the genomes and between the chromosomes. The St genome chromosomes were revealed to be most strongly conserved, with each of the 14 single-gene probes in the corresponding positions as described in the wheat chromosomes. Most of the Y genome chromosomes maintained the same synteny as the St chromosomes, except that 3Y was detected as having an additional hybridization site at 7S-1 in the interstitial regions of the short arm. Both 7S-1 and 7L-4 mapped on the long arm of 7Y, suggesting the pericentric inversion in this chromosome. The collinearity of the P genome chromosomes was more distorted. Besides the translocation between 2P and 4P, additional hybridization of 6L-1 in the subtelomeric region of the 1PL and additional site of 7S-1 in the telomeric regions of 2PS were revealed in P genome (Figs 3 and 4; Table 2).

**Table 2.** Localization of full length cDNA probes by FISH on chromosomes of *K.hirsuta*.

Wheat FISH probe name	FISH probe order on *Kengiliahirsuta*	Average distance from the centromere (μm) ± SE	FLcDNA, KOMUGI database	cDNA length, (bp, KOMUGI database)
1S-1	1St-S	0.36 ± 0.03	tplb0048d21	3487
	1Y-S	0.24 ± 0.01		
	1P-S	0.26 ± 0.01		
1L-1	1St-L	0.77 ± 0.02	tplb0013a02	5094
	1Y-L	0.61 ± 0.01		
	1P-L-1	0.92 ± 0.02		
2S-1	2St-S	0.42 ± 0.02	tplb0006k18	3777
	2Y-S	1.51 ± 0.01		
	4P-S	1.33 ± 0.03		
2L-1	2St-L	0.76 ± 0.01	tplb0007l09	3184
	2Y-L	0.83 ± 0.03		
	2P-L	1.54 ± 0.04		
3S-1	3St-S	0.71 ± 0.02	tplb0014n06	3256
	3Y-S-1	1.10 ± 0.01		
	3P-S	2.52 ± 0.05		
3L-1	3St-L	0.54 ± 0.02	AK336104	3860
	3Y-L	0.32 ± 0.02		
	3P-L	0.46 ± 0.03		
4S-6	4St-S	2.23 ± 0.03	plb0017g02	3191
	4Y-S	2.08 ± 0.02		
	4P-L	2.85 ± 0.07		
4L-4	4St-L	2.08 ± 0.03	AK335609	4790
	4Y-L	2.29 ± 0.03		
	2P-S-2	5.46 ± 0.02		
5S-1	5St-S	0.22 ± 0.01	tplb0016k09	3057
	5Y-S	0.26 ± 0.01		
	5P-S	0.90 ± 0.03		
5L-2	5St-L	2.20 ± 0.06	AK331808	4827
	5Y-L	2.60 ± 0.03		
	5P-L	3.72 ± 0.08		
6S-2	6St-S	0.79 ± 0.01	tplb0006a09	3703
	6Y-S	1.26 ± 0.01		
	6P-S	1.37 ± 0.03		
6L-1	6St-L	0.52 ± 0.01	tplb0009a09	3298
	6Y-L	0.70 ± 0.03		
	1P-L-2	6.18 ± 0.07		
	6P-L	1.69 ± 0.05		
7S-1	7St-S	1.00 ± 0.03	AK334430	4424
	3Y-S-2	1.45 ± 0.01		
	7Y-L	2.26 ± 0.03		
	2P-S-1	5.29 ± 0.04		
	7P-S	2.47 ± 0.04		
7L-4	7St-L	3.60 ± 0.06	tplb0007o14	3982
	7Y-L	3.56 ± 0.03		
	7P-L	6.55 ± 0.06		

**Table 3.** Chromosome measurements of *K.hirsuta*.

**Chromosome**	**Long arm (L)** ± **SE μm**	**Short arm (S)** ± **SE μm**	**Total Length (T=L+S)** ± **SE μm**	**Arm ratio (L/S)**	**Centromeric index (S/T) × 100**
1St	4.40 ± 0.96	3.14 ± 0.75	7.54 ± 1.60	1.40	41.67
2St	4.68 ± 0.62	4.08 ± 0.69	8.76 ± 1.25	1.15	46.62
3St	4.73 ± 0.43	3.09 ± 0.47	7.82 ± 0.87	1.53	39.52
4St	3.68 ± 0.40	2.98 ± 0.41	6.66 ± 0.80	1.23	44.78
5St	5.69 ± 0.78	2.67 ± 0.22	8.36 ± 0.98	2.13	31.95
6St	3.49 ± 0.54	3.11 ± 0.42	6.60 ± 0.90	1.12	47.08
7St	4.17 ± 0.70	3.88 ± 0.44	8.05 ± 1.14	1.07	48.24
1Y	3.47 ± 0.30	2.07 ± 0.16	5.54 ± 0.36	1.68	37.34
2Y	4.13 ± 0.34	3.43 ± 0.52	7.56 ± 0.83	1.20	45.40
3Y	3.83 ± 0.48	3.25 ± 0.32	7.08 ± 0.76	1.18	45.91
4Y	3.26 ± 0.21	2.46 ± 0.28	5.72 ± 0.47	1.33	42.96
5Y	4.60 ± 0.58	2.21 ± 0.33	6.81 ± 0.88	2.08	32.44
6Y	3.19 ± 0.25	2.92 ± 0.24	6.11 ± 0.46	1.09	47.77
7Y	3.97 ± 0.35	3.06 ± 0.50	7.03 ± 0.77	1.30	43.47
1P	7.16 ± 0.98	4.72 ± 0.80	11.88 ± 1.73	1.52	39.74
2P	7.90 ± 0.97	5.90 ± 1.18	13.80 ± 2.13	1.34	42.76
3P	7.67 ± 0.67	5.77 ± 0.73	13.44 ± 1.39	1.33	42.94
4P	7.60 ± 0.52	4.40 ± 0.40	12.00 ± 0.79	1.90	34.45
5P	8.80 ± 1.07	4.88 ± 0.56	13.68 ± 1.58	1.80	35.66
6P	6.30 ± 0.49	5.40 ± 0.71	11.70 ± 1.06	1.17	46.18
7P	7.51 ± 0.82	6.70 ± 0.67	14.21 ± 1.47	1.12	47.15

The same original cDNA probes were used for karyotype and chromosome structural analysis in *Agropyroncristatum* Gaertner, 1770, (2*n* = 14; P genome) (Said et al. 2018). Though fewer single-gene probes were used in *K.hirsuta* than in *A.cristatum*, chromosomal collinearity of the P genome across species was compared by shared common single-probes. Well-conserved single-probe hybridization positions were revealed in 3P, 5P and 7P across both species. Chromosome 1P in both *K.hirsuta* and *A.cristatum* showed conserved 1S-1 and 1L-1 hybridizations, but 1P in *A.hirsuta* included an additional hybridization 6L-1 in the subtelomeric region of the long arm. Chromosome 1P in *A.cristatum* was characterized as metacentric, whereas 1P in *K.hirsuta* was submentacentic. This indicates that 1P in *A.hirsuta* was structurally different from that in *A.cristatum*. Coincidentally, the reciprocal translocations between 2P and 4P were identified in both *K.hirsuta* and *A.cristatum*. The hybridization positions of single-gene probes 4L-4 and 2L-1 in 2P of *K.hirsuta* were corresponding to those in 2P of *A.cristatum*. Physical mapping more single-genes on 4P of *A.cristatum* revealed more complicated structural variations such as inversion. Though the limited single-gene probes were mapped in 4P of *K.hirsuta*, the single-gene probe 4S-6 in the long arm on 4P of *K.hirsuta* was accordant to those 4S-1, 4S-2, 4S-3, and 4S-4 in the long arm on 4P of *A.cristatum*. This observation indicates that although chromosomes 2P and 4P in *K.hirsuta* may be modified from those in *A.cristatum*, they are still considered to include an ancient common rearrangement. A paracentric inversion on the long arm of 6P was identified in *A.cristatum* (Said et al. 2018). However, the 6L-1 hybridization position in 6P of *K.hirsuta* was equivalent to the position of the homoeologous wheat chromosome This indicates no major chromosome arrangements in 6PL in *K.hirsuta*.

### Chromosome polymorphisms between different populations

**Figure 5.**
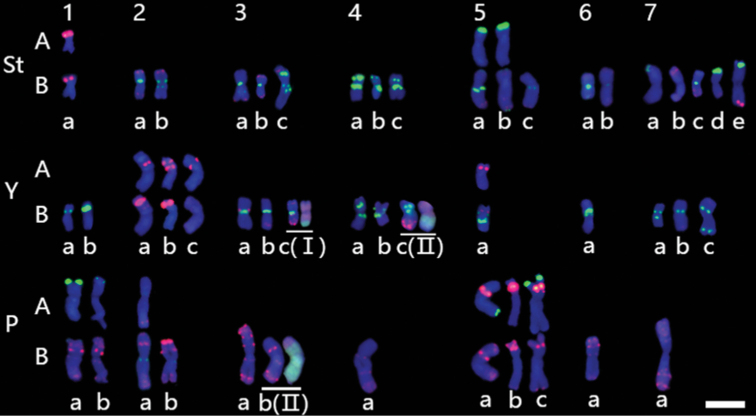
Molecular karyotypes of 29 *K.hirsuta* samples. The patterns of chromosomes were characterized by probe combinations A: 45S rDNA (green) and 5S rDNA (red); and B: pAs1 (red) and (AAG)_10_ (green). Different variants are annotated by different letters. The translocated chromosomes are underlined, and indicated by Roman numerals I–II. Scale bar: 10 μm.

Karyotyping was conducted on 29 individuals of *K.hirsuta* from 7 different populations, by using repetitive sequences as chromosomal landmarkers. Chromosomal homoeology were detenmined referring the above reference karyotype derived from HCZ population. The polymorphisms of each chromosome and the karyotype of each individual were well described (Fig. 5; Table 4). The results identified a total of 47 chromosomal variants in the 29 individuals (Table 4). The St genome showed the highest number of chromosome variants (19), whereas the Y genome had 16 and the P genome had 12. Chromosomes 1St, 5Y, 6Y, 4P, 6P, and 7P were the most stable, with identical variants across populations, while 7St was the most variable with more than 3 variants. The majority of individuals showed FISH patterns in the homozygous state, although some were in the heterozygous state (34.48%, ten of 29 plants; Table 4).

**Table 4.** Chromosome variants in *K.hirsuta*.

Population	Samples	St genome								Y genome								H genome							heterozygosis	
		1	2	3	4	5	6	7	1		2	3	4	5	6	7	1		2	3	4	5	6	7		
GCN	2	a	a	a	a	a	a	a	a		a	a	a	a	a	a	a		b	a	a	a	a	a		-
	4	a	a	a	ab	a	a	b	a		a	cT	a	a	a	a	a		a	a	a	b	a	a		+
	10	a	a	ab	a	b	a	c	a		a	cT	a	a	a	a	b		b	a	a	b	a	a		+
	12	a	a	a	a	a	a	b	a		b	cT	a	a	a	a	a		b	a	a	b	a	a		-
GCS	7	a	a	a	a	a	a	b	a		a	b	a	a	a	a	a		a	a	a	c	a	a		-
	10	a	a	a	a	a	a	b	b		a	b	b	a	a	a	a		a	a	a	c	a	a		-
	16	a	a	a	b	a	a	b	b		c	b	b	a	a	b	a		a	a	a	c	a	a		-
GMY	1	a	a	a	b	c	b	b	a		a	a	a	a	a	b	a		a	a	a	c	a	a		-
	3	a	a	a	b	a	a	a	a		a	a	b	a	a	b	a		a	a	a	c	a	a		-
	4	a	a	ab	b	a	b	a	a		a	a	a	a	a	b	a		a	a	a	c	a	a		+
	5	a	a	a	c	a	a	b	a		a	a	a	a	a	b	b		a	a	a	c	a	a		-
QL	1	a	a	a	c	a	b	a	ab		ac	a	a	a	a	b	b		a	a	a	c	a	a		+
	2	a	a	a	b	a	b	b	a		a	a	b	a	a	c	a		a	a	a	c	a	a		-
	3	a	a	ac	a	bc	a	b	a		a	a	a	a	a	ab	a		b	a	a	a	a	a		+
	9	a	a	a	a	ac	a	bd	a		ac	a	a	a	a	ab	ab		b	a	a	c	a	a		+
	6	a	a	a	a	c	ab	de	a		ac	a	a	a	a	b	b		a	a	a	c	a	a		+
HST	1	a	a	a	b	b	ab	b	a		a	a	a	a	a	b	a		a	a	a	c	a	a		+
	2	a	a	a	b	b	b	b	a		a	a	b	a	a	b	a		a	a	a	c	a	a		-
	8	a	a	a	a	a	b	b	a		a	a	a	a	a	b	a		a	a	a	c	a	a		-
XH	3	a	a	a	a	a	b	d	a		a	a	b	a	a	b	a		b	a	a	c	a	a		-
	5	a	a	a	a	a	b	d	a		a	a	b	a	a	b	a		b	a	a	c	a	a		-
	11	a	a	a	b	a	a	b	a		a	a	b	a	a	b	a		b	a	a	c	a	a		-
	10	a	a	a	a	b	a	d	a		a	a	b	a	a	b	a		a	a	a	c	a	a		-
	8	a	a	ab	a	b	a	d	a		a	a	b	a	a	b	ab		b	a	a	c	a	a		+
HCZ	1	a	a	a	a	a	b	b	a		a	b	cT	a	a	b	a		a	bT	a	b	a	a		-
	3	a	a	a	a	b	a	b	a		a	b	b	a	a	b	a		a	a	a	c	a	a		-
	4	a	b	a	a	a	a	a	a		a	b	b	a	a	b	a		a	a	a	c	a	a		-
	7	a	a	a	ac	b	a	b	a		a	b	b	a	a	b	a		a	a	a	c	a	a		+
	lb	a	a	a	a	a	b	b	a		a	b	cT	a	a	b	a		b	bT	a	b	a	a		-
No. of variants		1	2	3	3	3	2	5	2		3	3	3	1	1	3	2		2	2	1	3	1	1		
Total								19								16								12		10

**Note**: plus sign represents heterozygous karyotypes, minus sign represents homozygous karyotypes.

Most of the variants were characterized by the absence or presence of additional hybridization signals due to duplications, or deletions of repeats of pAs1 and (AAG)_10_, as well as the absence or presence of hybridizations of 45S rDNA (Fig. 5 5St c, 5P b). Moreover, translocations involved in 3Y, 4Y, and 3P were detected in a few individuals. A tentative pericentric inversion was identified in 5P (Fig. 5 5P a) in different populations.

Chromosomal structural variations were detected in 3Y, 7Y, 1P, 2P, and 4P by using single-gene probes in the individuals of the population HCZ. Furthermore, the chromosomal polymorphisms were revealed in above chromosomes across different populations. The polymorphisms of 1P were detected as the hybridization intensity variation of 45S rDNA in the terminal part of the short arm (Fig. 5 1P a and b), whereas those of 2P were detecet as an additional 5S hybridization in the intrcalary region of the long arm (Fig. 5 2P a and b). However, the structural variation detected by single-gene FISH was in the distal part of the long arm of 1P and the distal part of the short arm of 2P, respectively. No polymorphisms of 4P were detected between populations. It suggests that the chromosomal structural variation in 1P, 2P, and 4P might be species-specific. Since the polymorphism sites on the short arm of 3Y and those on the long arm of 7Y were corresponding to the arms involving structural variation detected by single-gene probes, it indicates the structural variations in 3Y and 7Y might be population-specific.

Although the number of samples was not the same in the different populations, the results indicate that the populations QL, HCZ, and GCN included more variants than the others (Table 4). In particular, one specific inter-genomic translocation variant (3Y c) with the P genome segment was identified in the population GCN; one specific reciprocal translocation (4Y c and 3P b) was identified in the population HCZ (Fig. 5); and the individuals of QL showed a high heterozygous state of 80% (Table 4).

## Discussion

The 45S rDNA products join with the 5S rDNA and the ribosomal proteins to make the ribosomes. The major sites of the 45s rDNA correspond to the NOR. The 45S rDNA sites of wheat were physically mapped in four different homoeologous groups, as noted in the short arms of 1A, 1B, 6B and 5D, and 7DL (Mukai et al. 1991). In *K.hirsuta*, 45S rDNA sites were mapped in the short arms of 5St, 1P, and 5P. The 45S rDNA sites in 1P and 5P detected in the present study were consistent with those in the P genome of *A.cristatum* (Said et al. 2018). 5S rDNA sites were physically mapped on chromosomes homoeologous group 1 (1AS, 1BS, 1DS) and group 5 (5AS, 5BS, and 5DS) (Mukai et al. 1990). In *K.hirsuta*, 5S rDNA sites were mapped in the short arms of 1St, 2Y, 5Y, and 5P. The detected 5S rDNA in the homoeologous group 2 in the Y genome was notably exceptional. Since the 5S rDNA site was absent in 1Y, the 5S site in 2Y might have been transferred from 1Yto 2Y by chromosomal rearrangements. The 45S rDNA, or 5S rDNA, or both detected in the short arms of homoeologous 5 of each genome suggests these are highly conservable across species. Moreover, the chromosomes of the homoeologous 5 group in each genome in *K.hirsuta* mostly showed the highest long arm to short arm ratio, consistent with the morphological characteristics of those in each genome in common wheat (Gill et al. 1991). This indicates that the chromosomal morphology of the homoeologous 5 across distant species in Triticeae is strongly conserved.

Four 45 rDNA sites were reported in accessions of the diploid species in *Agropyroncristatum* and *Agropyronmongolicum* (Keng, 1938) (Zhao et al. 2017). However, two 45S rDNA sites on 1P and 5P were mostly identified in this study. We also note the variability of the 45S rDNA in *K.hirsuta* in different populations. They were mostly identified as the presence and absence of the hybridization signals in the origins. The instability and copy number variation of rDNA were regarded to be particularly sensitive to genomic stresses, and acted as a source of adaptive response (Salim and Gerton 2019). Whether the detected intra population variations of 45S rDNA are associated with intra population differentiation needs further investigation.

Species in the tribe Triticeae are characterized by large genomes, the majority of which are repetitive DNA sequences (Flavell et al. 1974, 1986; Bennett et al. 1982). *K.hirsuta* has a large and complex genome as an allopolyploid species with three different genomes. Despite the high through-put sequencing techniques that are now available, exploring targeted genes from such large and complex genomes is still highly challenging. Since a few of crops in Triticeae are wholly sequenced, comparing genomics will be a shortcut for cloning targeted genes from wild relatives. For example, seed shattering is a key character for wild species domestication. The genes *Btr-1* and *Btr-2* controlling seed shattering were conservatively located in the short arms of homeologue 3 chromosomes in barley and wheat (Pourkheirandish et al. 2015; Zhao et al. 2019). In this study, the conserved synteny of chromosome 3St and 3P, and possible structural aberrations in the short arm of 3Y, were identified in *K.hirsuta*. This implies that the *Btr-1* and *Btr-2* might be more strongly conserved in the 3St and 3P chromosomes. Moreover, gene cloning may be facilitated by genome dissection during flow sorting or micro dissection (Tiwari et al. 2015; Said et al. 2018; Sheng et al. 2020; Soares et al. 2020). In this study, the chromosomes belong to the homoeologous group 3 were clearly identified. Furthermore, the target chromosomes can be labeled using more specific chromosomal markers, and tentatively isolated for gene cloning.

High karyotype variation was observed in the sympatric distributed Triticeae species *Elymusnutans* Griseb., 1868 (Dou et al. 2017). Though chromosomal polymorphisms were found in different individuals in *K.hirsuta* (47 in 29 individuals), there were far fewer variants than in *E.nutans* (100 in 27 individuals). In addition, in the related species *K.thoroldiana*, inter-genomic rearrangements affected by environmental factors were reported (Wang et al. 2012). However, only limited inter-genomic translocations were uncovered in the specific populations, suggesting that the karyotype of *K.hirsuta* is more stable than those of the related species. Nearly 40% of the individuals had heterozygous karyotypes in *K.hirsuta*, the frequency of which is higher than those in *E.nutans* (22.2%) (Dou et al. 2017). In the population of QL, the highest karyotype heterozygosis of 90% was detected in the present study. The higher heterozygosis indicates the frequencies of out-crossing in the populations in *K.hirsuta*. The origin of *Kengyilia* was suggested to be from natural amphiploids between the tetraploid *Roegneria* C. Koch, 1848 (StY genome) and diploid *A.cristatum* (P genome) (Yang et al. 1992). The StY species are highly self-crossing, but the P genome species are self-compatible as well as facultative allogamous (Dewey 1983). Whether or not the facultative allogamous system of P genome is functioning in *K.hirsuta* needs further investigation.
